# Ultrasound-indicated cerclage as a critical decision window in cervical insufficiency: a mini review of time-dependent risk interpretation

**DOI:** 10.3389/fmed.2026.1900226

**Published:** 2026-08-19

**Authors:** Yong-Jin Park, Moon-Il Park

**Affiliations:** 1 Department of Obstetrics and Gynecology, Dongtan Jeil Women's Hospital, Hwaseong, Republic of Korea; 2 Department of Obstetrics and Gynecology, Hanyang University Hospital, Seoul, Republic of Korea

**Keywords:** cervical insufficiency, cervical length, emergency cerclage, preterm birth, time-dependent risk, ultrasound-indicated cerclage

## Abstract

Ultrasound-indicated cerclage occupies an intermediate position between history-based prophylaxis and emergency or physical examination–indicated cerclage. Current guidelines generally define this intervention by obstetric history and cervical-length thresholds, but these categorical criteria do not fully capture the time-dependent nature of cervical insufficiency. This focused Mini Review synthesizes international guidelines, randomized and meta-analytic evidence, and recent observational studies to examine ultrasound-indicated cerclage as a critical decision window within a dynamic process of cervical remodeling and progressive structural failure. In this framework, sonographic cervical shortening represents a transitional phase in which risk has become clinically visible but may still remain modifiable. Evidence most consistently supports cerclage in selected high-risk singleton pregnancies with prior spontaneous preterm birth or mid-trimester loss and current cervical shortening, whereas benefit is less certain in low-risk populations with an incidentally detected short cervix or after progression to membrane exposure or advanced dilation. Interpretation is limited by heterogeneity in patient selection, cervical state, timing of intervention, and reporting of operative variables. Reframing ultrasound-indicated cerclage as a time-dependent decision window may help explain heterogeneous findings, improve interpretation of guideline differences, and support more precise patient selection. Future studies should report cervical-length trajectory, gestational timing, cervical phenotype, membrane relationship, inflammatory context, and transition to emergency operative states.

## 1 Introduction

Cervical cerclage has long been used as a preventive or salvage intervention for women at increased risk of mid-trimester pregnancy loss or spontaneous preterm birth. Current major guidelines frame cerclage primarily according to indication-based categories, including history-indicated, ultrasound-indicated, and emergency or physical examination–indicated cerclage (1–5). A recent comparative systematic review of international guidelines confirmed broad areas of agreement but also highlighted persistent variation in history thresholds, very short cervix, twin gestations, and physical examination–indicated cerclage (6). Similarly, a recent systematic review of double vs. single cerclage emphasized that cerclage outcomes are influenced by indication, operative context, and procedural heterogeneity (7).

This indication-based approach is clinically practical, but it provides only partial insight into how cervical failure evolves during an ongoing pregnancy. Women without prior obstetric history may develop progressive cervical shortening or present later with dilation or membrane prolapse, shifting intervention from prevention to salvage. Emergency cerclage outcomes depend on cervical state at presentation, including dilation, membrane exposure, infection risk, and technical feasibility (8).

Spontaneous preterm birth arises from a heterogeneous and multifactorial risk landscape rather than from a single fixed pathway (9). Biological studies of cervical extracellular matrix remodeling and clinical studies of cervical change suggest that cervical insufficiency is better understood as a dynamic process within the broader preterm-birth continuum, rather than as a static anatomic defect alone (10–12). This perspective is particularly relevant to ultrasound-indicated cerclage, because transvaginal cervical-length assessment can identify a phase in which cervical risk has become clinically visible but may still remain modifiable (13).

**Figure 1:**
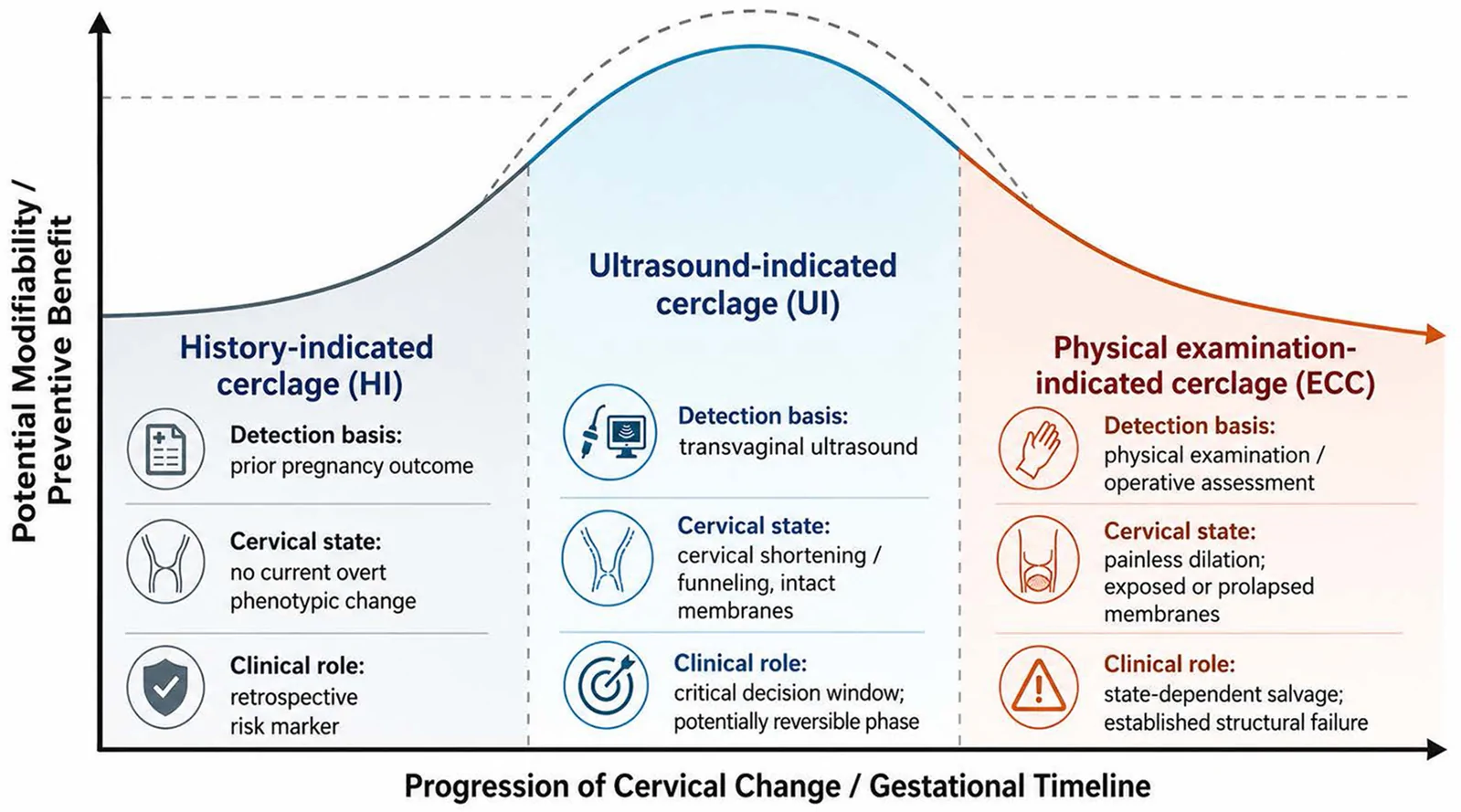
Detection basis, cervical state, and clinical role across the time-dependent cerclage continuum. History-indicated (HI) cerclage is based on prior pregnancy outcomes and functions as a retrospective risk marker; it does not necessarily indicate an active cervical phenotype in the current pregnancy. Ultrasound-indicated (UI) cerclage is triggered by transvaginal detection of cervical shortening or funneling while membranes remain intact and represents a potentially modifiable critical decision window. Physical examination–indicated cerclage, also referred to as emergency cerclage, is defined by overt cervical dilation and/or exposed or prolapsed membranes and functions primarily as state-dependent salvage intervention. The horizontal axis represents progression of cervical change across gestation, whereas the vertical axis reflects potential modifiability or preventive benefit. Differences in cerclage outcomes may therefore reflect not only indication labels, but also the basis of detection, cervical state, timing of intervention, and operative context. This figure is intended as a conceptual interpretive framework and does not constitute a validated clinical staging system or treatment algorithm.

Figure 1 distinguishes history-indicated, ultrasound-indicated, and physical examination–indicated cerclage by their basis of detection, cervical state, and clinical role. History-indicated cerclage reflects retrospective risk recognition rather than an active cervical phenotype in the current pregnancy. Ultrasound-indicated cerclage is triggered by transvaginal detection of cervical shortening or funneling while membranes remain intact, representing a potentially modifiable decision window. Physical examination–indicated cerclage is defined by overt cervical dilation and/or exposed membranes and functions primarily as state-dependent salvage.

Evidence syntheses indicate that cerclage benefit varies according to baseline risk, obstetric history, cervical-length threshold, and cervical state at intervention (14–18). Inflammatory and immune pathways contribute to the preterm-birth continuum, while recurrent preterm-birth patterns support heterogeneous rather than uniform mechanisms of recurrence (19, 20). Observational comparisons between ultrasound-indicated and emergency cerclage, as well as between elective and emergency cerclage, support the clinical relevance of gestational timing and cervical state at intervention, although interpretation is limited by differences in patient selection, cervical presentation, and operative management (21–24). Evidence remains most consistent in selected high-risk singleton pregnancies in which prior spontaneous preterm birth or mid-trimester loss is combined with current cervical shortening (15–17). Recent individual patient-data meta-analytic and randomized evidence has also informed ongoing debate regarding potential benefit in selected women without prior spontaneous preterm birth and with a very short cervix (14, 18).

This focused Mini Review was informed by a narrative search of PubMed/MEDLINE and major international guideline websites through June 2026. Search concepts included “cervical cerclage,” “ultrasound-indicated cerclage,” “short cervix,” “cervical insufficiency,” “emergency cerclage,” “physical examination–indicated cerclage,” and “preterm birth.” Priority was given to international guidelines, randomized trials, individual patient-data meta-analyses, systematic reviews, and recent observational studies relevant to patient selection, cervical-length thresholds, timing, cervical state, and pregnancy outcomes. Publications were selected for direct relevance to time-dependent interpretation of ultrasound-indicated cerclage; this was not a formal systematic review.

Taken together, these data suggest that ultrasound-indicated cerclage should be interpreted not simply as a cervical-length threshold, but as a time-dependent decision window between retrospective risk recognition and state-dependent salvage intervention. Table 1 summarizes international guideline recommendations, key eligibility criteria, management of short cervix without prior spontaneous preterm birth or mid-trimester loss, and selected special situations and evidence gaps relevant to ultrasound-indicated cerclage. This Mini Review examines ultrasound-indicated cerclage as a critical decision window, emphasizing gestational timing, cervical-length trajectory, membrane relationship, and transition to emergency operative states.

**Table 1:** International guideline recommendations, key indications, and evidence gaps relevant to ultrasound-indicated cerclage.

Guideline/source	Key recommendation for ultrasound-indicated cerclage	Short cervix without prior sPTB or mid-trimester loss	Special situations/evidence gaps	Decision-window implication
RCOG green-top guideline No. 75 (1)	Ultrasound-indicated cerclage is recommended when CL ≤ 25 mm before 24 weeks is identified in women with prior spontaneous preterm birth or mid-trimester loss.	Cerclage is not recommended for an incidentally detected CL ≤ 25 mm before 24 weeks in the absence of prior spontaneous preterm birth, mid-trimester loss, or another relevant high-risk factor.	Prior cervical trauma or congenital cervical anomaly may warrant individualized assessment. Emergency or physical examination–indicated cerclage may be considered up to 27+6 weeks in selected cases.	Distinguishes clinical indications, but does not explicitly frame progressive cervical shortening as a time-limited, potentially modifiable phase.
SMFM consult series #70 (2)	Addresses individuals without prior spontaneous preterm birth; cerclage is not recommended for CL 10–25 mm before 24 weeks in the absence of cervical dilation.	Cerclage is not recommended for asymptomatic singleton pregnancies with CL 10–25 mm before 24 weeks in the absence of cervical dilation.	No separate affirmative cerclage recommendation is provided for a cervix shorter than 10 mm without dilation. In twin gestations with cervical shortening, routine use of progesterone, pessary, or cerclage is not recommended outside a clinical trial.	Provides a clear pathway for short cervix without prior sPTB, but does not link history-based risk, sonographic change, and emergency presentation within one framework.
ACOG practice bulletin No. 142 (3)	Supports ultrasound-indicated cerclage when a short cervix is detected in women with prior spontaneous preterm birth or second-trimester loss.	Cerclage is not recommended solely on the basis of a short cervix in women without prior spontaneous preterm birth or second-trimester loss.	A single unexplained second-trimester loss may support history-indicated cerclage; specific thresholds for very short cervix are not provided.	Primarily history-based; cervical-length trajectory and timing of shortening are not specified as separate decision variables.
NICE NG25 (4)	Cerclage or vaginal progesterone may be considered when CL ≤ 25 mm between 16+0 and 24+0 weeks is combined with prior spontaneous preterm birth or mid-trimester loss.	Cerclage is not routinely recommended on the basis of CL ≤ 25 mm alone without prior spontaneous preterm birth or mid-trimester loss.	Cerclage may be considered when CL ≤ 25 mm is accompanied by prior cervical trauma or previous PPROM. Emergency cerclage may be considered between 16+0 and 27+6 weeks in selected cases.	Integrates obstetric history and current TVCL, but retains categorical recommendations rather than explicitly describing transitions in cervical state.
FIGO Good practice recommendations (5)	Recommends ultrasound-indicated cerclage for CL < 25 mm in women with one or more prior spontaneous preterm births and/or mid-trimester losses.	Evidence of benefit is insufficient for cerclage based solely on CL < 25 mm in the absence of prior spontaneous preterm birth, mid-trimester loss, or another relevant high-risk factor.	Benefit remains uncertain in women with cervical trauma or anomaly but no prior spontaneous preterm birth or mid-trimester loss. Possible benefit in twin gestations with CL < 15 mm is noted, but not as a firm recommendation.	Provides a concise international summary, but remains mainly threshold- and history-based rather than explicitly focused on cervical trajectory or timing of transition.

CL, cervical length; ECC, emergency or physical examination–indicated cerclage; PPROM, preterm prelabor rupture of membranes; sPTB, spontaneous preterm birth; TVCL, transvaginal cervical length; UI, ultrasound-indicated cerclage. Early sPTB refers to spontaneous preterm birth before 34 weeks' gestation. This table summarizes guideline recommendations and does not replace individualized clinical assessment.

## 2 Cervical insufficiency as a time-dependent condition

### 2.1 From a static defect to a time-dependent failure process

Cervical insufficiency has traditionally been described as a structural weakness of the cervix predisposing to painless mid-trimester dilation, pregnancy loss, or spontaneous preterm birth. This model shaped the rationale for history-indicated cerclage, in which prior adverse obstetric outcomes are interpreted as evidence of an underlying cervical defect (1–5). However, a static defect model does not fully account for the biological and clinical heterogeneity of cervical failure.

Cervical competence is better understood as a dynamic property that changes across gestation. Cervical extracellular matrix remodeling, inflammatory activation, mechanical loading, and pregnancy-specific risk factors interact over time, influencing whether cervical shortening remains stable, progresses, or transitions to dilation and membrane exposure (10, 19). Clinical studies also suggest that the rate and pattern of cervical change may provide prognostic information beyond a single cervical-length measurement (11). Recurrent preterm birth patterns further support heterogeneous rather than uniform pathways of risk recurrence (20).

This time-dependent interpretation has direct implications for cerclage. If cervical failure progresses along a trajectory, the expected benefit of cerclage should depend not only on the presence of a short cervix, but also on gestational timing, rate of cervical change, residual cervical structure, membrane status, and inflammatory context. Under this framework, ultrasound-indicated cerclage is clinically important because it identifies an intermediate phase in which cervical risk is no longer hidden, but structural failure may not yet have advanced beyond effective mechanical support (12).

### 2.2 Cervical phenotype beyond absolute cervical length

Absolute cervical length remains the main sonographic marker for risk stratification and the principal threshold variable in studies and guidelines addressing ultrasound-indicated cerclage. However, cervical length alone cannot fully describe the clinical state of the cervix. A single measurement does not distinguish a constitutionally short but stable cervix from a cervix undergoing rapid shortening or transitioning toward functional failure with funneling, dilation, or membrane exposure. Thus, the same cervical-length threshold may represent different biological and mechanical states depending on gestational age, prior risk profile, and the pattern of change over time.

Clinical studies suggest that the rate and pattern of cervical shortening may provide prognostic information beyond an isolated cervical-length value (11). A dynamic cervical phenotype incorporating cervical length, gestational timing, interval change, funneling, residual closed length, cervical consistency, and the relationship between the cervix and fetal membranes may better reflect the patient's position along the cervical failure trajectory. This phenotype-based interpretation also helps clarify why cerclage benefit is not uniform across all short-cervix populations: benefit is more consistently observed in selected high-risk women with prior spontaneous preterm birth or mid-trimester loss and current cervical shortening than in women with an incidentally detected short cervix alone (1–5, 14–18).

For ultrasound-indicated cerclage, the clinically relevant question is therefore not only whether the cervix is shorter than a predefined threshold, but whether the observed cervical state represents a modifiable phase before transition to emergency or physical examination–indicated cerclage. This interpretation provides the basis for viewing ultrasound-indicated cerclage as a critical decision window rather than merely a threshold-based intervention.

### 2.3 History-indicated cerclage as a retrospective risk marker

History-indicated cerclage represents the traditional form of risk-based cervical support. It is generally offered or considered when prior obstetric outcomes, such as recurrent mid-trimester loss or spontaneous preterm birth, are interpreted as evidence of underlying cervical vulnerability (1–5). This approach is clinically useful because it identifies high-risk women before cervical change becomes sonographically apparent. However, the risk signal is retrospective, derived from a previous pregnancy rather than from the evolving cervical state of the current pregnancy.

A history-based indication therefore functions as a proxy for presumed susceptibility rather than as a real-time marker of disease activity. Prior second-trimester loss or spontaneous preterm birth may reflect cervical insufficiency, but may also arise from infection, inflammation, uterine overdistension, placental disease, or other pathways within the preterm-birth continuum (9, 19, 20). Conversely, women without prior adverse outcomes may still develop progressive cervical shortening during the current pregnancy, particularly when risk is driven by newly expressed biomechanical, inflammatory, or iatrogenic factors (9, 10).

From a time-dependent perspective, history-indicated cerclage is best understood as upstream prevention based on retrospective risk enrichment. By contrast, ultrasound-indicated cerclage is triggered by contemporaneous cervical shortening, making latent risk clinically visible during the ongoing pregnancy. These two indications therefore represent different forms of risk recognition: history-indicated cerclage is based on retrospective inference, whereas ultrasound-indicated cerclage is based on real-time detection of a potentially modifiable cervical state. This distinction helps explain why the expected effect of cerclage may differ depending on whether it is applied before cervical change is detectable, during a sonographic transition, or after overt structural failure has occurred.

### 2.4 Ultrasound-indicated cerclage as the critical decision window

Ultrasound-indicated cerclage occupies an intermediate position within the clinical spectrum of cervical insufficiency. Unlike history-indicated cerclage, which is based on prior adverse obstetric outcomes, it is triggered by measurable cervical change in the current pregnancy. Unlike emergency or physical examination–indicated cerclage, it is generally performed before overt cervical dilation, exposed membranes, or advanced operative complexity are present (1–5, 8).

From a time-dependent perspective, the ultrasound-indicated setting represents the point at which cervical risk becomes visible but may still remain modifiable. Before cervical shortening is detected, risk may be latent or insufficiently specific to justify intervention. After progression to dilation or membrane exposure, the clinical problem shifts toward salvage, where prognosis depends heavily on cervical state, membrane status, infection risk, and technical feasibility (8, 21–24). Between these states lies the critical decision window, in which sonographic cervical shortening may identify patients transitioning from risk to structural failure before an advanced salvage state has developed.

This framework may help explain why cerclage benefit differs across short-cervix populations. In singleton pregnancies without prior spontaneous preterm birth, benefit remains uncertain overall for an incidentally detected short cervix, although recent individual patient-data meta-analytic and randomized evidence has renewed interest in selected women with a very short cervix (1–5, 14, 18). By contrast, randomized trials and meta-analyses more consistently support ultrasound-indicated cerclage in selected high-risk singleton pregnancies with prior spontaneous preterm birth or mid-trimester loss and current cervical shortening (15–17). Thus, cervical length alone is insufficient; benefit depends on baseline risk, cervical phenotype, and whether the current cervical state represents a potentially modifiable stage in the trajectory toward spontaneous preterm birth.

### 2.5 Emergency cerclage as state-dependent salvage intervention

Emergency cerclage, also referred to as physical examination–indicated cerclage, represents a later point along the cervical failure trajectory. Unlike history-indicated cerclage, which is based on retrospective risk, or ultrasound-indicated cerclage, which is triggered by sonographic cervical shortening, emergency cerclage is defined by the presenting cervical state. Cervical dilation, exposed or prolapsed fetal membranes, and the need for operative membrane reduction indicate that structural failure has already become clinically overt (1, 8).

This distinction is central to time-dependent risk interpretation. Once dilation and membrane exposure are present, the question is not simply whether cerclage is indicated, but whether it is technically feasible and biologically meaningful in the current operative state. Prognosis may depend on dilation, extent of membrane prolapse, membrane reducibility, residual cervical rim, gestational age, inflammatory or infectious status, and surgeon experience (8, 21–24). These variables are not fully captured by conventional indication labels, yet they may strongly influence latency and neonatal outcome.

Emergency cerclage should therefore be understood as a salvage intervention performed across heterogeneous operative states rather than as a uniform procedure. This does not mean that emergency cerclage lacks value; in selected cases, it may prolong pregnancy compared with expectant management (8). However, its role differs from ultrasound-indicated cerclage: emergency cerclage responds to manifest structural failure, whereas ultrasound-indicated cerclage aims to intervene before transition to an advanced salvage state. This distinction reinforces the importance of identifying progressive cervical shortening before dilation and membrane exposure occur.

## 3 Aligning intervention with cervical state

A time-dependent framework requires cerclage to be interpreted not only by indication category, but also by the cervical state at intervention. History-indicated, ultrasound-indicated, and emergency cerclage are useful clinical categories, but they also correspond to different points along a continuum of cervical risk: retrospective susceptibility, sonographic transition, and overt structural failure. Aligning intervention with cervical state may clarify when cerclage is preventive, when surveillance remains appropriate, and when the procedure has shifted toward salvage.

### 3.1 The transition from surveillance to intervention

The central challenge in ultrasound-indicated cerclage is determining when cervical surveillance should shift to intervention. Transvaginal ultrasound is often treated as a tool for identifying a short cervix, but in a time-dependent framework it detects an evolving cervical state before overt dilation, membrane exposure, or advanced operative complexity develops. The decision to intervene should therefore depend not on cervical length alone, but on whether cervical change suggests progression toward a clinically meaningful and still modifiable failure state.

In women with prior spontaneous preterm birth or mid-trimester loss, serial cervical-length surveillance refines retrospective risk during the current pregnancy. A cervix remaining longer than 25 mm before 24 weeks may suggest that prior risk has not translated into active cervical failure, whereas shortening to 25 mm or less identifies a subgroup in whom ultrasound-indicated cerclage is more consistently supported (1, 15–17). In women without prior spontaneous preterm birth, the evidentiary threshold for cerclage remains higher because an incidentally detected short cervix may not represent the same disease state as progressive shortening in a high-risk patient. However, recent individual patient-data meta-analytic and randomized evidence has renewed interest in selected women with a very short cervix (1–5, 14, 18).

A state-aligned approach interprets cervical length together with gestational age, interval shortening, funneling, residual closed length, early dilation, membrane relationship, and clinical or inflammatory context. This is the practical meaning of ultrasound-indicated cerclage as a critical decision window.

### 3.2 Explaining heterogeneous outcomes across UI cerclage studies

Heterogeneous outcomes across studies of ultrasound-indicated cerclage may reflect differences in patient selection, cervical state, and timing rather than uncertainty about the procedure alone. An incidentally detected short cervix in a low-risk woman, progressive shortening in a woman with prior spontaneous preterm birth, and early transition toward membrane exposure may all meet broad “short cervix” criteria, but they represent different points along the cervical failure trajectory. This helps explain why benefit is more consistently observed in selected high-risk singleton pregnancies than in unselected low-risk short-cervix populations, while recent individual patient-data meta-analytic and randomized evidence has renewed interest in selected women without prior spontaneous preterm birth and with a very short cervix (1–5, 14–18).

Study design and procedural heterogeneity further contribute to variability. Trials often define eligibility by cervical length and obstetric history, whereas observational studies may include mixed populations differing in gestational age, symptoms, membrane status, dilation, plurality, cervical trauma, or prior procedures (21–24). Evidence is particularly limited and heterogeneous in twin gestations, in which the effectiveness of ultrasound-indicated cerclage remains uncertain despite possible benefit in selected women with a very short cervix (25). When ultrasound-indicated and emergency cases are grouped together, poorer outcomes may reflect more advanced cervical failure at presentation rather than lack of cerclage efficacy. Operative variables, including technique, suture material, height of placement, number of stitches, adjunctive medication, and operator experience, may also influence outcomes but are inconsistently reported (7).

A time-dependent interpretation therefore suggests that ultrasound-indicated cerclage should be evaluated not only by cervical-length threshold, but by the modifiable cervical state in which it is performed. Future studies should report gestational age, cervical-length trajectory, membrane relationship, dilation, inflammatory markers, and operative details to clarify which patients are most likely to benefit within the critical decision window.

## 4 Discussion: guideline implications and future research

Current guidelines provide clinically useful categories for cerclage decision-making, but they remain structured mainly around obstetric history, cervical-length thresholds, and the presence or absence of overt cervical dilation (1–5). As summarized in Table 1, ultrasound-indicated cerclage is most consistently supported when current cervical shortening occurs in women with prior spontaneous preterm birth or mid-trimester loss, whereas guidelines are more cautious in women without prior history. This structure is evidence-based, but it does not fully operationalize the timing of cervical change, rate of shortening, or transition from a sonographic state to an emergency operative state.

A time-dependent framework may complement existing guidelines by interpreting history-indicated, ultrasound-indicated, and emergency cerclage as different points along a continuum of cervical risk. In this model, history-indicated cerclage reflects retrospective risk recognition, ultrasound-indicated cerclage identifies a potentially modifiable sonographic transition, and emergency cerclage represents state-dependent salvage after overt structural failure. This interpretation preserves guideline categories while clarifying how they relate to disease stage and modifiability.

Future studies should therefore report more than cervical length and obstetric history. Key variables include gestational age at diagnosis, cervical-length trajectory, interval shortening, funneling, residual closed cervical length, dilation, membrane exposure, inflammatory or infectious markers, plurality, prior cervical procedures, and operative details. Studies including emergency or physical examination–indicated cerclage should stratify cases by cervical state and operative feasibility, because these factors may strongly influence latency and neonatal outcomes (8, 21–24).

In selected cases near the boundary between ultrasound-indicated and physical examination–indicated cerclage, evaluation for intra-amniotic inflammation or infection may further refine patient selection. Recent studies have proposed invasive testing before cerclage and perioperative antimicrobial strategies, including broad-spectrum or multi-agent antibiotic regimens, to better characterize inflammatory or infectious contributors to cervical failure. However, these approaches should be interpreted cautiously because indications, diagnostic thresholds, antibiotic protocols, and operative states vary across studies.

Classification also requires precision. Recent correspondence has highlighted that pooling history-indicated, ultrasound-indicated, and emergency cerclage may obscure clinically meaningful heterogeneity in indication classification, cervical state, and operative context (26). Future research should avoid treating “short cervix” as synonymous with cervical insufficiency or analyzing “cerclage” as a uniform intervention without specifying indication, timing, cervical state, and operative configuration. Prospective validation of the decision-window model will require longitudinal studies evaluating whether cervical phenotype, timing, and rate of change improve prediction of benefit beyond cervical-length thresholds alone.

## 5 Conclusion

Ultrasound-indicated cerclage should be understood not only as a response to a cervical-length threshold, but as an intervention positioned within a time-dependent cervical failure process. Between history-indicated cerclage, which reflects retrospective risk recognition, and emergency cerclage, which responds to overt structural failure, ultrasound-indicated cerclage identifies a transitional phase in which cervical risk is detectable but may still be modifiable.

Reframing ultrasound-indicated cerclage as a critical decision window may help explain heterogeneous outcomes across studies by emphasizing differences in baseline risk, gestational timing, cervical phenotype, and operative state. Future research should prospectively evaluate whether cervical-length trajectory, timing of shortening, membrane relationship, inflammatory status, and operative details improve identification of patients most likely to benefit before progression to emergency salvage intervention.
